# Nucleolar Proteins and Non-Coding RNAs: Roles in Renal Cancer

**DOI:** 10.3390/ijms222313126

**Published:** 2021-12-04

**Authors:** Piotr Popławski, Joanna Bogusławska, Karolina Hanusek, Agnieszka Piekiełko-Witkowska

**Affiliations:** Centre of Postgraduate Medical Education, Department of Biochemistry and Molecular Biology, 01-813 Warsaw, Poland; piotr.poplawski@cmkp.edu.pl (P.P.); joanna.boguslawska@cmkp.edu.pl (J.B.); karolina.hanusek@cmkp.edu.pl (K.H.)

**Keywords:** renal cancer, renal cell carcinoma, RCC, nucleolus, snoRNA, lncRNA, microRNA, AluRNA, rRNA, ribosome biogenesis

## Abstract

Renal cell cancer is the most frequent kidney malignancy. Most RCC cases are classified as clear cell renal cell carcinoma (ccRCC), characterized by high aggressiveness and poor prognosis for patients. ccRCC aggressiveness is defined by classification systems based on changes in morphology of nucleoli, the membraneless substructures of nuclei. The latter act as the sites of ribosome biogenesis as well as the hubs that trap and immobilize proteins, preventing their action in other cellular compartments. Thereby, nucleoli control cellular functioning and homeostasis. Nucleoli are also the sites of activity of multiple noncoding RNAs, including snoRNAs, IGS RNA, and miRNAs. Recent years have brought several remarkable discoveries regarding the role of nucleolar non-coding RNAs, in particular snoRNAs, in ccRCC. The expression of snoRNAs is largely dysregulated in ccRCC tumors. snoRNAs, such as SNHG1, SNHG4 and SNHG12, act as miRNA sponges, leading to aberrant expression of oncogenes and tumor suppressors, and directly contributing to ccRCC development and progression. snoRNAs can also act without affecting miRNA functioning, by altering the expression of key oncogenic proteins such as HIF1A. snoRNAs are also potentially useful biomarkers of ccRCC progression. Here, we comprehensively discuss the role of nucleolar proteins and non-coding RNAs in ccRCC.

## 1. Introduction

Renal cell carcinoma (RCC) is the most common subtype of renal cancers. The vast majority of RCC cases are classified as clear cell renal cell carcinoma (ccRCC), encompassing 80–90% of diagnosed tumors [1,2]. The other RCC types include papillary renal cell carcinoma (pRCC, up to 18.5% RCC cases), chromophobe renal cell carcinoma (chRCC, <5%), and clear cell papillary renal cell carcinoma (ccpRCC, 3–4%). The rare types of renal malignancies include collecting duct carcinoma (less than 1% of diagnosed tumors), hereditary leiomyomatosis and renal cell carcinoma-associated renal cell carcinoma, renal medullary carcinoma, as well as several other uncommon kidney tumors [3,4]. The prognosis for ccRCC patients with early-stage tumors is good, with 5-year survival reaching 90% [5]. Most ccRCC cases are diagnosed accidentally, e.g., during ultrasonography performed due to other indications. As a consequence, up to 30% of ccRCC cases are diagnosed at advanced stages. A further 25–30% of patients develop metastatic ccRCC (mccRCC) during the years following the diagnosis [6,7]. In contrast to patients with localized disease, the prognosis for metastatic patients is poor. mccRCC in the vast majority of cases is practically an incurable disease. Current treatment options, offered to metastatic patients, include inhibitors of tyrosine kinases (TKIs), inhibitors of mTORC signaling, as well as recently introduced inhibitors of immune control checkpoints (e.g., nivolumab). Despite the initial effectiveness, most mccRCC patients inevitably develop resistance to these modern therapies with disease progression and death following. As a consequence, the life of patients with mccRCC treated with targeted therapies can be prolonged to up to 40 months [6,7].

One of the key features of ccRCC cells are changes in the morphology of the nucleoli, which are small nuclear substructures involved in the biogenesis of the ribosomes. Notably, recent studies showed that nucleoli can greatly contribute to the functioning of cells by immobilization of proteins, thereby preventing their activity in other cellular compartments [8,9]. Nucleoli are also the sites of actions of numerous non-coding RNAs (ncRNAs), including snoRNAs (small nucleolar RNAs), AluRNAs (transcribed from repetitive elements in human genome), IGS RNAs (intergenic space RNAs) or miRNAs (microRNAs). Aberrant expression of these ncRNAs, in particular snoRNAs, is frequently observed in cancers, contributing to enhanced proliferation, migration, invasion or chemoresistance [10].

## 2. Clear Cell Renal Cell Carcinoma: The Molecular Background

The key molecular features of ccRCC include inactivation of VHL tumor suppressor, which occurs in up to 90% of cases, as well as frequent mutations of the PI3K/Akt pathway, SETD2 (H3K36 methyltransferase), and genes encoding the elements of the SWI/SNF complex (PBMR1, ARID1A, and SMARCA4) [11,12]. Recent studies revealed that proliferation of ccRCC cells is also driven by reprogrammed tumor cell metabolism. These changes include aberrances in the TCA cycle, the pentose phosphate pathway (PPP), metabolism of amino acids and fatty acids. As in other cancer types, the glycolytic pathway of ccRCC cells is adapted to convert pyruvate into lactate even under normoxic conditions. This so-called Warburg effect enables synthesis of ATP with concomitant enhanced production of substrate molecules (nucleotides, amino acids, fatty acids) required as ‘building bricks’ during intense proliferation of cancer cells [13,14]. The accumulation of lactate produced during anaerobic glycolysis results in local acidosis. This acidic environment triggers rapid nucleolar localization of VHL [9]. In normal proximal tubule cells, VHL acts as an E3 ubiquitin protein ligase, which enables proteasomal degradation of hypoxia-inducible factors (HIF1A and HIF2A) under normoxic conditions. During hypoxia, VHL-dependent HIF degradation is disabled, enabling the HIFs to activate the transcription of target genes (e.g., VEGF, PDGF) and, thus, prevent harmful effects of oxygen deprivation [11]. Regardless of the nucleolar trapping mechanism, frequent mutations of *VHL* gene inactivate its function in ccRCC tumors. This leads to persistent activation of the HIFs and stimulation of angiogenesis, supporting tumor growth. Thus, the nucleolar trapping of VHL under the acidic environment constitutes an additional mechanism, preventing the HIFs’ degradation. Remarkably, this effect is independent of oxygen supply [9]. The same study suggested that sequestration of proteins could be a general mechanism by which the nucleoli could influence functioning of the cell. Further studies showed that, indeed, the nucleoli can trap and immobilize proteins (including VHL) by binding them to nucleolar non-coding RNAs [8]. It can be hypothesized that the nucleolus and its ncRNAs could be used as targets for therapeutic interventions to interfere with the mechanisms supporting metabolic reprogramming, growth and proliferation of cancer cells.

## 3. The Nucleolus

The nucleolus is a membraneless compartment of the nucleus, organized into three zones: the fibrillar center (FC), the dense fibrillar component (DFC), and the granular component (GC). The nucleolus is formed during the process of ribosome biogenesis and its key role is providing the molecular environment for transcription and processing of rRNA, which is assembled into ribosomal subunits. The latter are exported from the nucleolus to cytoplasm to form mature ribosomes [15,16]. The vast majority of rDNA (ribosomal DNA) is transcribed by RNA polymerase I (RNApol I) at the border between FC and DFC, giving the 47S pre-rRNA, which is co-transcriptionally processed and cleaved into 18S, 5.8S, and 28S mature rRNA. The fourth type of rRNA, 5S rRNA, is transcribed outside the nucleolus by RNA polymerase III (RNApol III), and subsequently imported with snoRNAs to participate in rRNA processing [15,17].

The initial concept of nucleoli functioning only as the sites of ribosome biogenesis was changed following proteomic studies that revealed that more than 30% of nucleolar proteins are not involved in ribosome biogenesis, suggesting novel, non-canonical functions of the nucleolus [18]. Indeed, it was discovered that nucleoli control cellular functioning and homeostasis by acting as hubs that trap and immobilize proteins, preventing their action in other cellular compartments [8,9]. The nuclear sequestration of proteins can be induced by different stimuli, including acidosis, serum starvation and DNA damage or ribosomal stress [8]. Remarkably, all these types of stress signals are present in cancer cells, suggesting the particularly important role of nucleoli in cancer.

The nucleolus has recently emerged as a key controller of cellular homeostasis, including proliferation and metabolism [19]. The size and morphology of the nucleolus are tightly associated with the cell cycle, while ribosome biogenesis is coordinated with the rates of protein synthesis and cellular metabolism of growing cells. Uncontrolled rDNA transcription and misregulation of RNApol I activity may contribute to enhanced proliferation of cancer cells [15]. Furthermore, it was shown that multiple oncogenic and tumor suppressive proteins affect functioning of the nucleolus by, respectively, enhancing (the oncogenes) or suppressing (tumor suppressors) the activity of RNApol I. Remarkably, several drugs inhibiting RNApol I and several other drugs targeting nucleolar proteins entered clinical trials following promising results of preclinical studies [15,20].

## 4. Nucleolar Small and Long Non-Coding RNAs in Cancer

The major group of ncRNAs that reside in the nucleolus are snoRNAs. They participate in the maturation of pre-rRNA by guiding the primary transcript to take its proper position in large ribonucleoprotein (RNP) complexes [21]. snoRNAs are typically 60–300 nt-long non-coding RNAs encoded by host genes, which are located in introns of protein-coding and non-coding genes. In the case of the latter, the non-coding genes consisting of exons and introns are transcribed, with the following posttranscriptional processing, leading to the generation of transcripts consisting of only intronic sequences. These long non-coding RNAs are called small nucleolar RNA host genes (SNHG) [22]. snoRNAs are classified into two groups, based on sequence differences. The box C/D snoRNAs contain specific sequence motifs called box C (RUGAUGA) and D (CUGA), while H/ACA snoRNAs bear box H (AnAnnA) and ACA motif. Box C/D snoRNAs participate in 2′-O methylation of rRNA, while H/ACA snoRNAs assist in pseudouridylation of rRNA. snoRNAs can be processed into smaller RNA species, called snoRNA-derived RNAs (sdRNAs), which are 18–22 nt long and may function in a manner similar to miRNAs or participate in alternative splicing [21].

Recent studies revealed that expression of snoRNAs is altered in tumors. A pancancer study demonstrated general upregulation of snoRNAs in 13 cancer types and proved that enhanced expression of SNORD46 promotes proliferation, migration and invasion of cancer cells [23]. The other cancer-promoting snoRNAs include SNORA42 in colorectal cancer (CRC) and lung cancer, SNORD126 in hepatocellular carcinoma and CRC, SNORA23 in pancreatic cancer, and many others [24,25]. Notably, altered expression of snoRNAs triggers activation of key cancer-associated signaling pathways involving PI3K/Akt, MAPK/ERK, and TGF-β [25]. Silencing of oncogenic SNORA42 attenuates tumorigenicity of lung cancer cells in vitro and in vivo [24]. 

Initial studies indicated that the nucleolar structure is mainly regulated by the process of ribosome biogenesis. Recent data provided evidence that ncRNAs play a significant role in nucleolar morphology and function [8,26]. The fact that the nucleoli are devoid of the surrounding membrane enables their rapid assembly and dispersion, reflecting changes in transcriptional activity. The nucleoli function as liquid-phase droplets that emerge from the nucleoplasm in a process of liquid–liquid phase separation (LLPS). This process is largely dependent on specific nucleolar proteins such as nucleolin (NCL), fibrillarin (FBL), and nucleophosmin (NPM1). It was shown that short transcripts encoded by intronic Alu repeat elements transcribed by RNAPol II interact with NCL and NPM1. Depletion of AluRNAs leads to dispersion of nucleoli and a reduction in pre-rRNA synthesis, while enhanced expression of AluRNAs results in increased size of the nucleoli and enhanced synthesis of pre-rRNA [26]. These effects are attributed to a specific subset of AluRNAs that are generated through alternative splicing of pre-mRNAs of protein coding genes that contain intronic Alu repeat elements. This splicing generates short AluRNAs that form a scaffold for nucleolin and nucleophosmin that enable NOR (nucleolus organizer region) formation due to their interactions with UBF protein [27].

**Figure 1:**
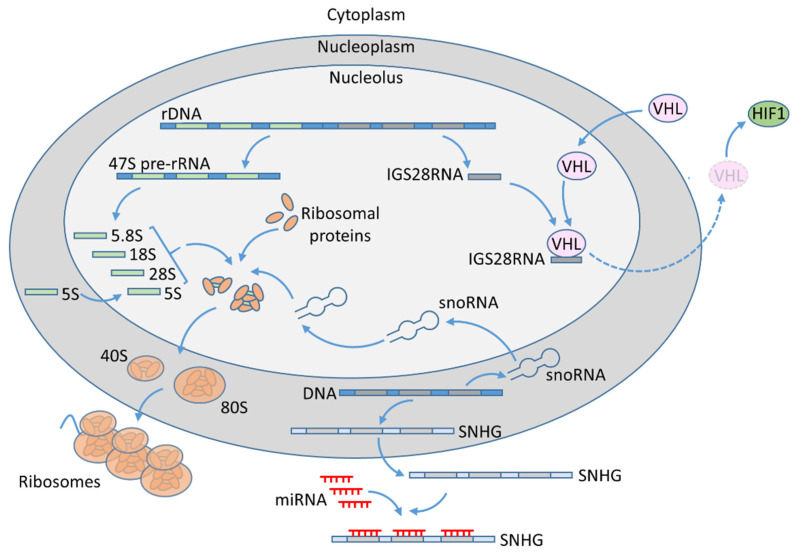
The role of nucleoli in ccRCC. Ribosome biogenesis: In the nucleolus, RNApol I generates 47S pre-rRNA, which is processed into 5.8S, 18S, and 28S rRNA. 5S rRNA is transcribed by RNApol III in the nucleoplasm and imported into nucleolus. The rRNA transcripts are processed and assembled together with ribosomal proteins, resulting in 40S and 60S ribosomal units. Ribosome maturation is assisted by snoRNAs, which are transcribed by RNApol II in the nucleoplasm from intronic sequences. SNHG transcripts are exported into cytoplasm where they can act as miRNA sponges, preventing their suppressive actions on target genes, including tumor-promoting MDM4, HIF1A, COL11A1, RUNX2, WNT2, and IGF1-R (see text for details). IGS28RNA is specifically expressed under acidic conditions in hypoxia. It binds and traps VHL tumor suppressor in the nucleolus, preventing its actions in the cytoplasm. As a result, VHL-mediated ubiquitination and proteasomal degradation of HIF1 are attenuated, leading to its stabilization.

Audas et al. [8] found that intergenic space (IGS) regions of genomic regions encoding rRNA produce ncRNAs that interact and immobilize proteins harboring nucleolar detention sequence (NoDS) in response to environmental stress [8]. In particular, the study confirmed interactions of VHL and POLD1 proteins with IGS28RNA. The IGS28RNA is specifically expressed under acidic conditions in hypoxia, and its interaction with VHL leads to retention of the latter in the nucleolus (Figure 1). These findings are particularly interesting regarding the fact that VHL is a key tumor suppressor inactivated in ccRCC. The other nucleolar ncRNAs that are involved in nucleolar sequestration of proteins include IGS16 and IGS22 RNAs, which are induced by heat shock and interact with HSP70, and IGS20, which binds HDM2. These results indicate that the identified group of ncRNAs enables the nucleolus to regulate cellular homeostasis by sequestration of proteins in response to specific environmental stimuli.

Several papers reported the existence of miRNAs in the nucleolus. Politz et al. showed that various microRNAs are enriched in the nucleoli of rat myoblasts [28]. Nucleolar miRNAs were also detected in human cancer cell lines HeLa and MCF7 [29]. The significance and role of nucleolar miRNAs is unknown. It was suggested that miRNAs could be imported to the nucleolus to interact with their target transcripts. The formed miRNA–mRNA complex could then be exported to the cytoplasm where regular miRNA-mediated suppression of expression could be executed [30]. It was also suggested that miRNAs may mediate interactions of rDNA with Ago2 protein, which regulates the rate of rRNA synthesis [31]. Li et al. identified 11 miRNAs that were enriched in the nucleoli of HeLa cells [32]. The sequestration of miRNAs in nuclear compartments may affect the functionality of small RNAs. Lemus-Diaz et al. demonstrated that miR-103a-3p, which is highly expressed in HeLa cells, displays only minimal functionality because it is mainly confined in the nucleus [33]. Eckstein et al. performed co-staining of miRNAs and their targets in prostate tissues and showed that miR-375 is specifically detected in the nucleoli of metastatic samples, indicating that nucleolar sequestration may play an important role in the regulation of microRNA functionality in cancer [34].

## 5. ccRCC and the Nucleolar Proteins

The irregularities in nucleolar morphology (size and shape) correlate with the progression of ccRCC tumors. In 1982, based on these observations as well as other nuclear parameters, Fuhrman et al. proposed a grading system for classification of RCC tumors, which enabled the prediction of distant metastases following nephrectomy [35]. The links between ccRCC pathology and nucleolar dysfunction were further underscored by the more recent introduction of the novel ISUP/WHO (International Society of Urological Pathology/World Health Organization) grading system, which is based on the assumption that nucleolar hypertrophy is associated with patient outcome [36]. 

Several studies suggested that targeting of nucleolar proteins may be possibly useful in the treatment of ccRCC patients. Altered expression of ribonucleoproteins (RNPs), including NOP56, correlates with poor survival of ccRCC patients [23]. Silencing of nucleophosmin 1 attenuates the viability of ccRCC cells [37], while chemical compounds that target nucleophosmin 1 inhibit the growth of the A498 ccRCC cell line [38]. The expression of DDX31 nucleolar protein is increased in ccRCC tumors and correlates with poor survival of ccRCC patients. DDX31 knock-down inhibits proliferation, growth, and viability of ccRCC cells, and triggers their apoptosis [37]. Treatment of a metastatic RCC patient who failed from treatment with prior TKI with AS1411, a DNA aptamer targeting nucleolin, resulted in an 84% reduction in tumor burden and inhibition of tumor progression for 2 years [39]. 

ccRCC proliferation is driven by specific disturbances of cellular metabolism, including aberrances in glycolysis, the pentose phosphate pathway, TCA cycle, as well as metabolism of amino acids and nucleotides [13,14,25]. Furthermore, ccRCC tumors of different grades have distinct metabolic profiles [40]. These findings as well as the knowledge that ccRCC tumor grades are defined by changes in nucleolar morphology suggest that changes in expression of nucleolar genes are functionally associated with altered metabolism of ccRCC tumors. In support of this hypothesis, it was shown that enzymes involved in the metabolic pathways that are reprogrammed in ccRCC (glycolysis, PPP, metabolism of amino acids and nucleotides) can localize to the nucleolus under specific conditions, such as the presence of HIV-1 Tat protein or Insulin/IGF1-PI3K signaling [41,42]. Gizak et al. found that phosphoglycerate mutase (PGAM), a glycolytic enzyme converting 3-phosphoglycerate to 2-phosphoglycerate, accumulates in the nucleolus where it interacts with 40S and 60S ribosomal proteins. Apparently, the nucleolar PGAM localization is crucial for proper functioning of the nucleoli, as PGAM silencing disturbs nucleolar structure and inhibits synthesis of RNA. PGAM depletion results in a decrease in the mitotic index of squamous cell carcinoma cells, suggesting that it could be a target for anticancer therapies [42]. Interestingly, PGAM is overexpressed in ccRCC tumors and its high expression is associated with tumor stage and size, suggesting correlation with tumor progression [43].

These findings indicate that nucleoli can indeed affect key cancer-related pathways in ccRCC cells and contribute to the development and progression of ccRCC.

## 6. ccRCC and the Nucleolar Non-Coding RNAs

The major group of nucleolar ncRNAs are snoRNAs. Initial transcriptomic studies revealed that differential expression of snoRNAs allows for distinguishing different subtypes of renal cell cancer [44]. A more recent pancancer study revealed that the expression of snoRNAs is generally upregulated in ccRCC tumors [23]. The study demonstrated that ccRCC tumors express a specific subgroup of snoRNAs, different from those characteristic of other cancer types [23].

Recent years have brought multiple papers reporting altered expression of snoRNA host genes in ccRCC (Table 1). Most of these studies demonstrated that upregulation of SNHG transcripts correlates with poor prognosis for patients. For instance, Yang et al. analyzed the expression of 20 SNHG in the publicly available ccRCC transcriptomic data of The Cancer Genome Atlas (TCGA). They found subsets of upregulated (SNHG1, GAS5, SNHG3-8, SNHG11, SNHG12, SNHG15-17, SNHG20, SNHG22 and SNHG25) and downregulated (SNHG9, SNHG10, DANCR and SNHG14) SNHGs. The increased expression of SNHG3 and SNHG15 correlated with poor survival of patients [45].

The expression of six of them (SNORA2, SNORD12B, SNORA59B, SNORA70B, SNORD93 and SNORD116-2) correlated with poor survival of patients [46]. Based on their expression, the risk score was built, which predicted overall survival (OS) and recurrence-free (RFS) survival of ccRCC patients. Furthermore, the risk score correlated with advanced TNM stage, higher Fuhrman grade, as well as lower hemoglobin level. The expression of the six snoRNAs was also evaluated in patients’ sera and confirmed their utility, indicating that the snoRNAs could serve as a non-invasive ccRCC biomarker [46].

The mechanisms behind altered expression of snoRNAs in ccRCC are largely unknown. Two studies demonstrated CpG methylation correlated with expression changes of snoRNA host genes (SNHG3, SNHG15, SNORD12B, and SNORD93). This suggests that epigenetic dysregulation could contribute to disturbed expression of snoRNAs in ccRCC [45,46].

These widespread alterations of SNHGs expression observed in ccRCC contribute to tumorigenesis and cancer progression. In general, two types of SNHG-mediated protumorous actions can be observed. SNHG transcripts can act as endogenous competing RNAs (or ‘sponges’), which bind microRNAs with complementary nucleotide sequences, preventing their regulatory actions on target genes. On the other hand, SNHG can regulate the functioning of key transcription factors, resulting in transcriptional reprogramming of genes involved in cancerous progression. In particular, several studies reported enhanced expression of SNHG12 in ccRCC tumors, which correlated with poor prognosis for patients, promoted viability, proliferation, migration and invasion, and inhibited apoptosis in vitro, as well as promoted ccRCC tumor growth in vivo [47,48,49]. All those studies reported that SNHG12 acted as a sponge for miRNAs, preventing them from inhibiting the expression of target genes. Wu et al. found that SNHG12 directly targeted and bound miR-129-5p, resulting in suppressed miRNA expression. This in turn resulted in upregulation of miR-129-5p target gene, MDM4, a suppressor of p53 activity [47]. In the study of Chen et al., SNHG12 acted as a sponge for miR-199a-5p, preventing it from binding to HIF1A transcript, resulting in enhanced expression of HIF1A protein [50]. Xu et al. found that SNHG12 acted as a sponge for miR-200c-5p, resulting in its downregulation. The study revealed that miR-200c-5p is a direct regulator of COL11A1, involved in cell adhesion and remodeling of extracellular matrix. SNHG12-mediated suppression of miR-200c-5p upregulated COL11A1, leading to apoptosis inhibition as well as increased viability [48]. Finally, SNHG12 was found to act as a sponge for miR-30a-3p, resulting in upregulation of RUNX2, WNT2 and IGF1-1R, contributing to ccRCC tumor progression [49].

This miRNA ‘sponging’ effect seems to be a general mechanism of SNHGs in cancers (Figure 1). For instance, SNHG1 functions within the feedback regulatory circuit involving miR-137. Specifically, miR-137 directly targets SNHG1 transcript to suppress its expression, while SNHG1 is a negative regulator of miR-137. In RCC cell lines, reduced expression of miR-137 contributes to SNGH1 upregulation and promotion of its oncogenic actions in RCC tumors. Experiments in ccRCC cell lines revealed that SNHG1 stimulated proliferation and invasion, as well as epithelial-to-mesenchymal transition (EMT). Those effects were reflected by observations in ccRCC patients. The expression of SNHG1 is increased in RCC tumors and correlates with poor survival of patients [51]. Interestingly, these alterations may also contribute to the immune evasion of RCC tumors. Increased expression of SNHG1 in RCC tumors results in suppression of miR-129-3p, and upregulation of its target, STAT3. This in turn results in STAT3-mediated transcriptional activation of PD-L1, which acts as a ligand for PD1 receptor expressed by T CD8+ lymphocytes. As a consequence, the proliferation of T CD8+ cells, as well as the release of cytokines (IFN-γ, TNF-α, and IL-2), is suppressed in the ccRCC microenvironment, resulting in diminished cytotoxicity of T CD8+ cells. In vivo, these SNHG1 effects result in attenuated infiltration of ccRCC tumors by T CD8+ lymphocytes, leading to enhanced tumor growth [52].

Another procancerous snoRNA host gene acting in ccRCC tumors is SNHG3. Its expression is increased in ccRCC tumors and correlates with poor prognosis for patients. SNHG3 promotes ccRCC progression both in vitro and in vivo by binding and repressing miR-139-5p, leading to the upregulation of its target, TOP2A. This in turn results in EMT induction, enhanced proliferation, migration, and invasion of ccRCC cells [53]. Similarly, Wu et al. found that enhanced SNHG4 expression correlates with tumor progression in RCC patients. Increased SNHG4 expression stimulated proliferation, migration, and invasion inhibited apoptosis of RCC cells in vitro and promoted tumor growth in vivo. These effects were mediated by suppressed expression of miR-204-5p, which was sponged by SNHG4, resulting in enhanced expression of miR-204-5p target, RUNX2 [54]. Likewise, enhanced expression of SNHG5 in ccRCC tumors leads to suppression of miR-363-3p, thereby resulting in upregulation of its target, Twist1. The latter is a transcription factor that stimulates the expression of genes encoding metalloproteinases MMP2 and MMP9, which modulate extracellular matrix. Altogether, the SNHG5-miR-363-3p-Twist1-MMP2/MMP9 axis promotes invasion and inhibits apoptosis of ccRCC cells [55]. 

An interesting SNHG is GAS5, which encoded ten C/D box snoRNAs and a H/ACA snoRNA SNORD103. The GAS5-encoded C/D box snoRNAs (SNORD81, SNORD47, SNORD80, SNORD79, SNORD78, SNORD44, SNORD77, SNORD76, SNORD75, and SNORD74) are predicted to regulate 2’O-methylation of rRNA, thereby facilitating RNA folding and interaction with ribosomes, while SNORDA103 contributes to pseudouridylation [56]. GAS5 acts as a competing endogenous RNA repressing miR-21, which results in upregulation of the microRNA target, SOX5. In ccRCC tumors, the expression of GAS5 is suppressed, leading to the upregulation of miR-21 and decrease in SOX5, resulting in sorafenib resistance [56,57]. 

As aforementioned, snoRNAs can also act without affecting miRNA functioning. Zhao et al. found that high SNHG6 expression was associated with clinical features of ccRCC malignancy and acted as a prognostic biomarker. Increased SNHG6 expression stimulated proliferation, migration, and invasion, as well as glucose metabolism of ccRCC cells in vitro. In vivo, enhanced SNHG6 expression promoted tumor growth and lung metastasis in mice. Those effects were mediated by YBX1, an activator of HIF1A translation. SNHG6 interacted with YBX1 and the formed complex stimulated HIF1A translation, resulting in increased HIF1A protein expression, thereby stimulating the growth, glucose uptake and migration of ccRCC cells [58]. An interesting tumor-promoting effect of SNHG15 was described by Du et al. [59]. They found that enhanced SNHG15 expression in ccRCC tumors stimulates migration and invasion by regulating NF-kB signaling pathway. Specifically, SNHG15 triggered transport of NF-kB to the nucleus, resulting in transcriptional activation of key EMT transcription factors, Snail1, Slug, and ZEB1. This in turn resulted in the induction of EMT, and enhanced motility and invasion of ccRCC cells [59].

**Table 1:** snoRNAs with altered expression in ccRCC tumors when compared with controls.

snoRNA	Alteration of Expression; Correlation with Prognosis (If Known)	Ref.
NHG9, SNHG10, DANCR and SNHG14	Downregulated	[45]
SNORA70F, SNORA2, SNORD116-24, SNORD116-4, SNORD116-2, SNORD116-26, SNORD116-1, SNORD116-27, SNORA80B	Downregulated	[45]
SNHG1, GAS5, SNHG3-8, SNHG11, SNHG12, SNHG15-17, SNHG20, SNHG22 and SNHG25	Upregulated; SNHG3: poor; SNHG15: poor	[45]
SNORD63 and SNORD96A	Upregulated	[60]
SNORD99, SNORD60, SNORD104, SNORA73B, SNORD123, SNORD63, SNORA16, SNORA71A, SNORD93, SNORA71C, SNORA7, SNORD124, SNORD12B, SNORD117, SNORA53, SNORA59B, snoZ196, SNORD17, SNORD15B	Upregulated; poor (for the signature consisting of six snoRNAs (six of them (SNORA2, SNORD12B, SNORA59B, SNORA70B, SNORD93 and SNORD116-2)	[46]
SNHG1	Upregulated; poor	[51]
SNHG4	Upregulated; poor	[54]
SNHG6	Upregulated; poor	[58]
SNHG12	Upregulated; poor	[47,48,49,50]

## 7. Nucleolar Non-Coding RNAs in Other Types of Kidney Cancers

Reports on nucleolar non-coding RNAs in renal malignancies other than ccRCC are scarce. The aforementioned study [46], describing the six-snoRNA signature predicting poor prognosis for ccRCC patients, also evaluated its value in chromophobe and papillary RCC. The signature had low sensitivity and specificity of survival prediction in these cancer types [46]. Zhang et al. demonstrated increased expression of SNHG12 in papillary renal cell carcinoma [61]. An interesting study was published by Lawrie et al. [44] who performed microarray analysis to evaluate the expression of snoRNAs and scaRNAs (small Cajal body-specific RNAs) in pRCC, ccRCC and clear cell papillary renal cell carcinoma (ccpRCC). The latter is a rare renal malignancy representing a mixture of features of other renal cancer types, resulting in a diagnostic challenge. Lawrie et al. reported that the expression patterns of snoRNAs and scaRNAs allowed for differentiation among the three analyzed renal cancer types. Unfortunately, they did not specify which of the ncRNAs were aberrantly expressed, nor provided validation of their results; therefore, it is difficult to conclude on the diagnostic utility of nucleolar ncRNAs for ccPRCC and pRCC [44].

## 8. Conclusions and Future Perspectives

ccRCC is the most aggressive subtype of renal cancer. This aggressiveness correlates with pleomorphism of the nucleoli, which have recently brought attention as the hubs regulating cellular homeostasis and metabolism. The proteins and non-coding RNAs related to the nucleolar functioning are often dysregulated in cancers, including ccRCC. Recent studies showed that snoRNAs are particularly often aberrantly expressed in ccRCC, contributing to tumorous progression. snoRNAs may also represent clinically useful biomarkers of ccRCC progression. 

Despite these interesting discoveries, multiple questions still remain to be answered. In particular, the links between nucleolar pleomorphism and snoRNAs dysfunction in ccRCC need to be experimentally verified. The current WHO/ISUP system of classification of ccRCC tumor grades is based on microscopic observations of nucleoli. This means that it is highly dependent on the experience of the pathologist, resulting in the risk of high inter-observer differences. This lack of objectivity may affect clinical decisions. It might be hypothesized that alterations of non-coding RNAs observed in ccRCC tumors may contribute to the morphological changes of the nucleoli. This hypothesis is supported by observations of AluRNAs whose depletion leads to dispersion of nucleoli and a reduction in pre-rRNA synthesis [26]. Interestingly, observations performed on yeast showed that loss of trimethyl guanosine synthase I (Tgs1p), which regulates snoRNAs’ post-transcriptional modifications, leads to changes in nucleolar morphology [62]. These data may suggest that changes in snoRNA expression/functioning may reflect the morphological changes of nucleoli, observed in ccRCC of different tumor grades. This, in turn, may suggest that snoRNAs may be potentially useful as objective biomarkers of ccRCC aggressiveness, supporting clinical decisions. This hypothesis should be verified by future studies.

Another important issue is the lack of studies on the role of snoRNAs and ccRCC chemoresistance. Furthermore, the role of nucleolar miRNAs and IGS RNA in ccRCC is unknown. Finally, the role of nucleolar ncRNAs in other renal cancer types is largely unexplored. Undoubtedly, the field of nucleoli-related proteins and non-coding RNAs is open for future studies and, hopefully, will bring novel discoveries aiming at a better understanding of RCC pathology and the development of new methods of therapy.
